# A Randomized Controlled Trial of Moderate-Intensity Circuit Band Resistance Exercise Program Improve Aerobic Exercise Ability in Older Adults

**Published:** 2019-05

**Authors:** Hyun-Min CHOI, Tai-Hyung KIM

**Affiliations:** 1. Department of Sports Sciences, GwangJu University, Gwangju, Korea; 2. Graduate School of Technology Management, KyungHee University, Yongin, Korea

## Dear Editor-in-Chief

As time passes, the growing old gradually drives to decrease physical structures and functions, and age-related changes reduce physical activities in daily life for old adults. However, they have not enough physical activities to maintain and improve their physical functions even they recognize it (1). Especially, while the aerobic exercise decreases body fat, heart rate in steady state, and blood pressure (2), the resistance exercise is more effective for improving basal metabolic rate, and muscular strength (3). Furthermore, circuit training is a program to execute an aerobic exercise and resistance exercise simultaneously (4). However, the most exercising type for this circuit training is dependent on the large weight machine and so it has a disadvantage to be available only at the specific place (5). Therefore, this study aimed to investigate the effect of circuit training by using an elastic band, and furthermore to provide the basic information for developing an exercise intervention program in terms of improving physical function of old adults.

This study was a designed a randomized controlled trial (RCT) in 2016. The elastic band resistance exercise (EBRE) program was performed for 60 min per one exercise and three times a week during 12 wk. The subjects of this study (n=200) were recruited for older than 65 yr without exercising habits and psychiatric problems in Korea. Of these 200 participants, we were excluded having medical problem (n=70), history of hypertension (n=40), heart disease (n=18), and refuse to participate (n=29). Ultimately, 43 participants (15 men, 28 women) were included in the current study.

Bioelectric impendence analysis (BIA) was used to measure height, body weight, fat mass (FM), fat-free mass (FFM), waist-hip ratio (WHR) which is an indirect index of abdomen fat rate, and body mass index (BMI). The test of resting blood pressure was measured sitting position and twice at interval of 5 min and the average was used. After measuring the blood pressure in a stead state, a blood flow volume (BFV) and a vascular diameter (VD) on the right brachial artery of subjects were measured by using 12 MHz B-mode Liner Transducer and Pulse Wave Doppler. Time average mean velocity (TAMV) was measured from the blood vessel and the artery at the angle of 60 degrees. And BFV was calculated by using measured TAMV and the VD.

There were significant differences between groups in weight (*P<*0.05), fat mass (*P<*0.05), BMI (*P<*0.05), and WHR (*P<*0.05) except for fat-free mass (Table 1). The exercise group may have an effect on cardiovascular responses than the control group. There were significant differences between groups in decreasing SBP (*P*<0.01), DBP (*P*<0.01), and MAP (*P*<0.01), and increased VD (*P<*0.05), and BFV (*P<*0.05).

**Table 1:** Comparison of body composition and cardiovascular responses between exercise and control group in EBRE program

***Variables***	***Groups***	***Pre M±SE***	***Post M±SE***	***Δ(%)***	***F***	***P***
*Body composition*						
BW (kg)	EG (n=21)	62.6 ± 2.0	60.9 ± 2.0^#^	–2.6	3.949	.046^*^
	CG (n=19)	59.5 ± 1.9	59.1 ± 1.9	–0.6		
FFM (kg)	EG (n=21)	24.0 ± 1.1	24.0 ± 1.1	0.0	0.471	.498
	CG (n=19)	26.8 ± 1.5	27.2 ± 1.4	1.5		
FM (kg)	EG (n=21)	18.0 ± 1.5	16.9 ± 1.4^#,†^	–6.2	3.869	.047^*^
	CG (n=19)	20.9 ± 1.5	20.5 ± 1.0	–2.2		
BMI (kg/m^2^)	EG (n=21)	24.3 ± 0.7	23.8 ± 0.6^#,†^	–2.1	3.858	.049^*^
	CG (n=19)	25.6 ± 0.8	25.6 ± 0.6	0.2		
WHR (%)	EG (n=21)	0.89 ± 0.0	0.86 ± 0.0^#,†^	–3.4	7.218	.012^*^
	CG (n=19)	0.87 ± 0.0	0.88 ± 0.0	1.2		
*Cardiovascular response*						
SBP (mmHg)	EG (n=21)	128.0 ± 2.7	122.0 ± 1.7^#,†^	–4.4	7.855	.009^*^
	CG (n=19)	133.0 ± 2.8	138.0 ± 3.4	3.6		
DBP (mmHg)	EG (n=21)	79.0 ± 1.7	76.0 ± 1.8^#,†^	–3.9	9.262	.005^*^
	CG (n=19)	79.0 ± 2.9	83.0 ± 2.1^#^	5.5		
MAP (mmHg)	EG (n=21)	95.0 ± 1.7	91.0 ± 1.4^#,†^	–4.1	10.689	.003^*^
	CG (n=19)	97.0 ± 2.7	101.0 ± 2.0^#^	4.7		
VD (cm)	EG (n=21)	0.39 ± 0.0	0.42 ± 0.0^#^	10.0	5.167	.030^*^
	CG (n=19)	0.42 ± 0.0	0.42 ± 0.0	–1.1		
BFV (ml/min)	EG (n=21)	0.03 ± 0.0	0.06 ± 0.0^#,†^	109.9	6.789	.014^*^
	CG (n=19)	0.03 ± 0.0	0.04 ± 0.0	16.3		

Values were expressed as mean (M) ± standard error (SE).

* *P*<0.05;

# significantly different vs. Pre-test,

† significantly different vs. CG.

The EBRE program which can be actually applicable in the preventative aspect should be composed for improving the aerobically exercise ability of old adults in local society and must be supplied to health and senior citizen center. In addition, the EBRE program to maintain and improve in daily life is necessary with applying towards elderly for healthy aging.
